# Supplementary material for: Complex‐centric proteome profiling by SEC‐SWATH‐MS
Source: Mol Syst Biol. 2019 Jan 14;15(1):e8438. doi: 10.15252/msb.20188438 (PMC6346213; doi:10.15252/msb.20188438)
Supplement: Supplementary file 7 — Dataset EV6 [file MSB-15-e8438-s007.zip › feature_plots_bioplex/Q9H5K3.pdf]

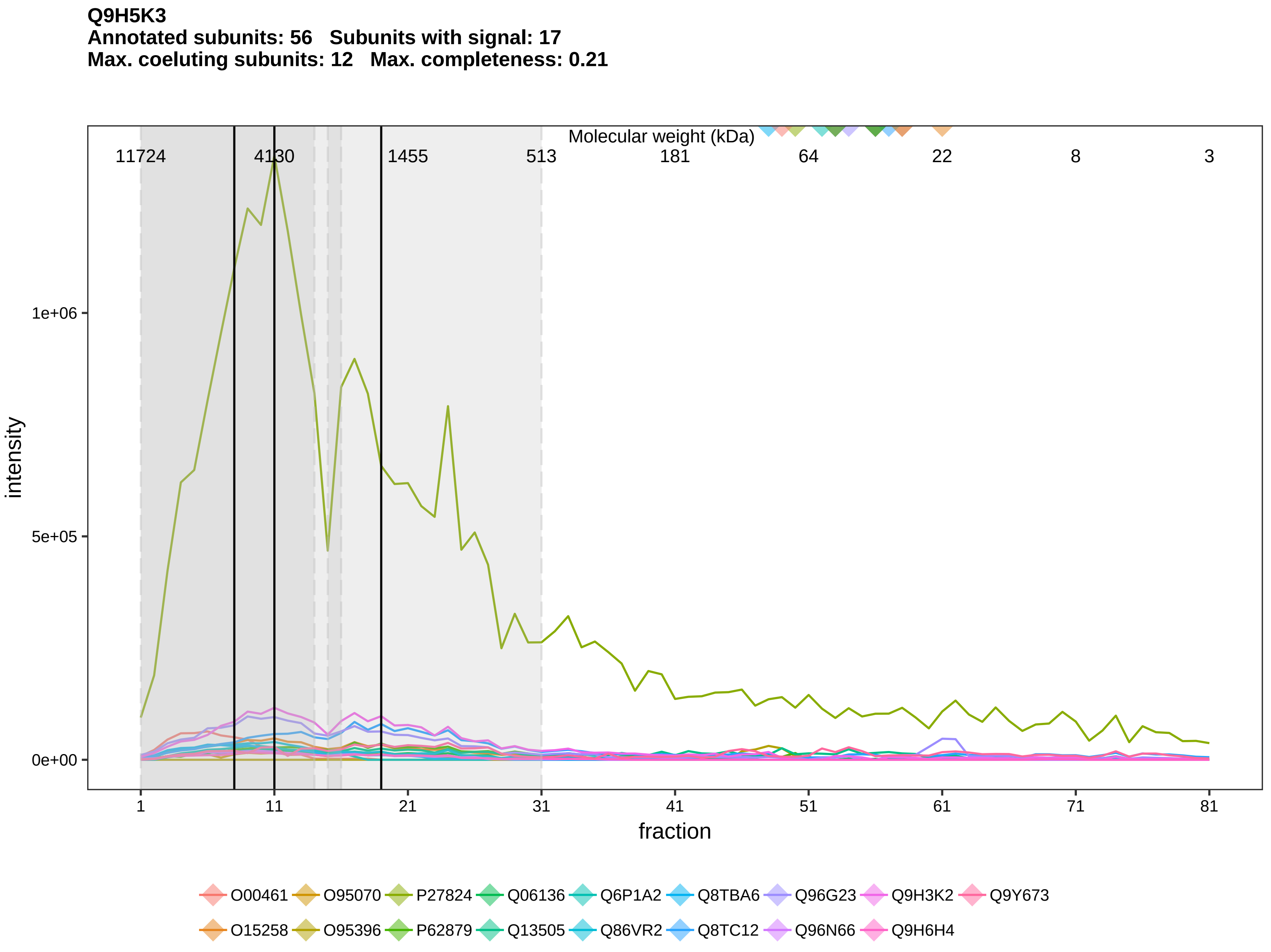

Q9H5K3
Annotated subunits: 56 Subunits with signal: 17
Max. coeluting subunits: 12 Max. completeness: 0.21
Molecular weight (kDa)
11724
4130
1455
513
181
64
22
8
3
intensity
1e+06
5e+05
0e+00
fraction
1
11
21
31
41
51
61
71
81
O00461
O95070
P27824
Q06136
Q6P1A2
Q8TBA6
Q96G23
Q9H3K2
Q9Y673
O15258
O95396
P62879
Q13505
Q86VR2
Q8TC12
Q96N66
Q9H6H4
